# Praising the police, avoiding the station: gendered differences on police–public relations in Kuwait

**DOI:** 10.3389/fsoc.2025.1700697

**Published:** 2025-10-29

**Authors:** Nasser AlSabah, Kieren Aris, Susan Nembhard, Malak Al-Rasheed

**Affiliations:** 1 John Jay College of Criminal Justice, CUNY Graduate Center, New York, NY, United States; 2 Kuwait National Police, Ministry of Interior, Kuwait City, Kuwait; 3 Department of Sociology and Social Work, Kuwait University, Kuwait City, Kuwait

**Keywords:** gender inequality, patriarchy, gender norms, police and public relations, perceptions of the police, Kuwait, Middle East

## Abstract

**Introduction:**

Women's engagement with key public institutions in the Middle East remains deeply shaped by patriarchal social structures, particularly within the male-dominated criminal justice system. While this dynamic is broadly acknowledged, its implications for police–community relations remain underexplored in empirical research. This study addresses this gap by examining how gender influences public perceptions of, and willingness to cooperate with, the police in Kuwait—a context where cultural norms and institutional hierarchies continue to shape women's interactions with law enforcement.

**Methods:**

Data were drawn from a nationally representative survey conducted by Kuwait's National Police, encompassing responses from 1,050 members of the public. The analysis assessed how gender influences three central outcomes: (1) trust in the police, (2) perceptions of officer behavior, and (3) willingness to cooperate with law enforcement. Both bivariate and multivariate statistical techniques were employed to detect significant differences and correlates across gender, while controlling for demographic variables such as age, marital status, and educational background.

**Results:**

Bivariate analyses revealed a striking paradox: women reported more favorable evaluations of police behavior than men, yet demonstrated significantly lower willingness to cooperate with law enforcement. Subsequent multivariate models confirmed that age, marital status, and education were significant predictors of women's attitudes toward the police. These findings indicate that social and cultural dynamics—beyond gender alone—substantially influence women's perceptions and behaviors within the justice system.

**Discussion and conclusion:**

This study offers the first quantitative evidence from the Middle East illustrating how gender inequality shapes police–community relations. Women's comparatively positive assessments of police conduct, coupled with their greater reluctance to cooperate, reveal a deeper tension embedded within patriarchal norms that limit women's agency and engagement with formal institutions. These results underscore the need for gender-sensitive policing strategies and institutional reforms aimed at enhancing women's trust and participation in the justice process. Policymakers and reform advocates should draw on these insights to strengthen women's empowerment, civic confidence, and equitable access to justice across the region. Future research should expand this inquiry by investigating additional cultural and institutional mechanisms that facilitate or impede women's meaningful inclusion in law enforcement interactions.

## 1 Introduction

One must first recognize the institutional and cultural context shaping women's interactions with the state to understand the dynamics of police-community relations in Kuwait. The national police force remains an overwhelmingly male profession, immediately establishing a gendered power dynamic (Strobl, 2008). This structural reality amplifies the effect of deeply ingrained social norms that act as barriers to reporting crime (Charrad, 2011). Concepts like family honor and a woman's reputation serve as powerful social controls that discourage reporting crimes. These controls are linked to informal gender norms that assert women should avoid interacting with male-led institutions, including the police. This inhibits women's practical ability to seek justice, fueled by fears of social stigma within their communities and undermines their ability to engage in the justice system proactively. This study confronts this issue directly through its focus on gender, investigating how these dynamics influence women's willingness to cooperate with law enforcement.

Kuwait, an oil-rich nation located in the Gulf Cooperation Council (GCC), depends heavily on its National Police for public safety and national security (AlSabah and Al-Rashidi, 2025). Established in 1938, the national police force has become a cornerstone of Kuwaiti society. While earning recognition for its emphasis on community policing (Almutairi, 2013), the institution also reflects the nation's traditionally gendered social structures. Unlike Western models, Kuwait requires aspiring commanding officers to undergo a rigorous 4-year program at the Saad AlAbdulla Police Academy, preparing cadets for leadership roles, supplemented by a 6-month program for degree holders (AlSabah et al., 2025). This structured approach has contributed to Kuwait's low crime rate, ranking it 31st globally on the Global Peace Index (2025).

Despite these achievements, Kuwait still faces challenges in police-public relations, a common issue in policing across many jurisdictions. Particularly of interest are gender dynamics associated with these struggles, ones that are heavily intertwined with cultural dynamics tied to the Arab region. Investigating these gendered dynamics provides valuable insights into Middle Eastern perspectives on police-community interactions and cultural influences on law enforcement attitudes, an area that has been scarcely researched. Furthermore, Kuwait's socio-political context, including cultural norms such as honor, family reputation, and traditional gender roles, offers a unique base for case studies. The patriarchal structures of law enforcement^1^, a predominantly male institution, and their associated societal gender norms^2^ create potential barriers to justice. This dynamic highlights why an empirical examination is crucial for assessing women's true access to justice.

### 1.1 Police and public relations

While academic research in criminal justice has extensively examined public perceptions of law enforcement, emphasizing their critical role in effective and equitable policing (Wu and Sun, 2010). Trust in the police is a critical component of effective policing and public cooperation (Boda and Medve-Bálint, 2017; Hevi et al., 2022), and serves as a key indicator of whether all citizens, regardless of gender, have equal access to state protection. The public's willingness to cooperate can impact how effectively law enforcement carries out its duties and how successful it is (Hamm et al., 2017).

Scholarly work suggests that public trust in law enforcement is significantly influenced by factors such as procedural justice (Bolger and Walters, 2019), community policing initiatives (Hevi et al., 2022), and the perceived integrity of law enforcement (Nalla et al., 2018). Negative experiences with police often erode public trust and law enforcement legitimacy, fostering unfavorable attitudes detrimental to effective policing (Hamm et al., 2017; Sunshine and Tyler, 2003). Comparative research related to public sentiment on law enforcement has also showcased significant effects cultural factors play, serving as potential catalysts to positive/negative attitudes among the public (Liu et al., 2020; Tankebe et al., 2019). According to Areh et al. (2007), women generally report higher satisfaction with police services than men, though they often perceive officers as being dismissive of their opinions. In this article, perspectives toward policing in Kuwait will be examined, particularly through a gender-based lens aimed at uncovering how societal constructs of gender inequality influence attitudes toward the police.

### 1.2 Gender, culture, and policing in the Middle East

Research on law enforcement and the criminal justice system in the Middle East remains sparse. While previous studies have examined public relations in the context of economics, politics, and social practices (Alanazi, 1996; Almutairi and Dashti, 2019; Kirat, 2005; Kruckeberg, 1996), there has been little to no research focusing on these themes in relation to criminal justice and law enforcement. This gap highlights the need for further investigation into the region's unique dynamics of gender inequality as they manifest in state-citizen interactions.

Arab societies are deeply influenced by patriarchal structures, which significantly shape gender roles and contribute to persistent gender inequalities across various sectors. These dynamics are further complicated by cultural norms that emphasize respect for male authority. As a result, women's perceptions of male-dominated police forces may differ from those observed in Western contexts, where gender equality is more advanced. Although there are no direct studies examining the influence of gender disparities on public attitudes toward law enforcement in the Middle East, existing research offers valuable insights into related themes. For instance, Al-Kandari et al. (2022) study, guided by the Spiral of Silence Theory, examined public opinions on women's involvement in Kuwait's police force. Results indicated that while support for female empowerment positively predicted acceptance of female law enforcement officers, fears of social isolation and religious intolerance strongly discouraged women from joining. These attitudes significantly impact women's participation in policing, posing a challenge to normalizing and promoting diversity in the profession.

The concept of hegemonic masculinity is also deeply embedded in police culture, emphasizing traits such as toughness and aggression that can alienate women and discourage their cooperation. In Turkey, (Ekşi 2019) explored initiatives aimed at dismantling hegemonic masculinity within law enforcement through cultural and organizational reforms. These measures, which included promoting diversity and adopting alternative policing approaches, were found to mitigate the dominance of masculine ideals in policing. Male dominance in law enforcement perpetuates the view of policing as a masculine profession, subjecting women within the force to scrutiny and reinforcing cultural norms that favor male authority in interactions with the public.

It should be noted that recent shifts in policing within the region have aimed to mitigate gender dynamics described in the literature above. In Saudi Arabia, the Ministry of Interior's creation of dedicated women's security roles marked an important milestone in increasing female participation in public safety. During the 2020 Hajj season, women police officers joined Makkah's security force for the first time (Arab News, 2020). In the United Arab Emirates, gender inclusion has become more institutionalized. Training institutions like the Dubai Police Academy and Rabdan Academy have introduced gender-inclusive programs in fields such as forensic science, cybersecurity, and crisis management. In Kuwait, 2025 marked the largest graduation of female officers in its history, with 171 female officers being sworn in (KUNA, 2025). While these initiatives signal a regional trend toward gender inclusion, their impact on public perception remains unclear. As scholarly work emphasizes, simply adding women to male-dominated institutions does not automatically dismantle patriarchal dynamics (Strobl, 2008). Therefore, a key question this study addresses is whether these top-down policy shifts translate into greater willingness among female citizens to engage with police—a critical gap in the existing regional literature.

A comprehensive study by Chu (2018) offers valuable insights into these institutional mindsets. The research, which surveyed 622 police officers in the United Arab Emirates (344 men and 278 women), revealed notable gender-based differences in perceptions of female leadership. Female respondents were significantly more likely to view female supervisors as competent, whereas male respondents were markedly less willing to accept serving under female leadership. This disparity may reflect the influence of entrenched masculinity within the law enforcement profession. These internal dynamics have external consequences: they influence the composition of the police force and public perceptions of police legitimacy, systematically marginalizing female representation in managerial roles (Charrad, 2011; Strobl, 2008).

This gendered perception of law enforcement is not arbitrary; research consistently shows that gender shapes the very criteria by which police legitimacy is judged. Some women may favor traditional forms of law enforcement over service-oriented ones (Hawdon, 2008). Lee et al. (2024) found that female suspects reported more favorable opinions of law enforcement, attributing this to more lenient treatment by police, compared with their male counterparts. Similarly, Sunshine and Tyler (2003) found that fairness and respect were particularly important for women, who often evaluate police through a social and relational lens (Hinds and Murphy, 2007; Sargeant et al., 2016). Media portrayals of gender and crime can also disproportionately shape women's views of police legitimacy as well (Graziano and Gauthier, 2018; Jackson et al., 2012).

While this literature establishes that gender matters, it has rarely been applied to the patriarchal context of the Middle East, where cultural norms surrounding honor and male authority may create unique barriers to women's cooperation with police. This study addresses that critical gap by providing the first empirical analysis of how gender shapes perceptions of police in Kuwait, offering insights into the cultural dynamics that may facilitate or hinder women's equal access to the justice system.

### 1.3 Current study

This cross-sectional study aims to investigate gender-based discrepancies in opinions on law enforcement in Kuwait while also examining the determinants of women's perceptions of police and police practices. Survey data from 1,050 members of the Kuwaiti public were analyzed to address these elements. Based on the patriarchal structures that shape police-citizen encounters in Kuwait, we hypothesize a paradoxical relationship in public perceptions: (H1) Women will report significantly more favorable views of police interactions than men, but will also report a significantly lower willingness to cooperate with the police. Additionally, we anticipate that women's social position will moderate their ability to navigate these structures: (H2) Among women, factors that increase social autonomy (such as age and higher education) will be positively associated with favorable opinions of law enforcement. In contrast, factors that reinforce traditional roles (such as marital status) will be negatively associated with female's willingness to cooperate with them. The findings are expected to provide novel insights into public opinions on law enforcement in the Middle East, contributing to a broader understanding of the cultural dynamics that influence criminal justice systems in the region. This study aims to provide a foundation for future research and policy initiatives targeting gender-specific challenges in policing and public trust.

## 2 Materials and methods

### 2.1 Data

The data for this study was sourced from Kuwait's Public Security division, a key sector within Kuwait's National Police that engages most frequently with the public (AlSabah, 2025). Data collection took place during the first half of 2022 through a collaborative effort between the Public Security division and researchers from the Department of Sociology and Social Work at Kuwait University. This partnership aimed to advance research on community policing in Kuwait. The study will primarily use survey data.

The survey, conducted with a sample of Kuwaiti citizens (*n* = 1,050), was designed to capture public perceptions of the police across various themes associated with police-public relations. Data collection employed a combination of convenience and snowball sampling methods. Physical surveys were distributed to college students at Kuwait University, who were encouraged to involve friends and family in completing the questionnaires. The survey also gathered demographic information from respondents, including age, gender, marital status, job status, education level, and province of residence. Questions were scored on a 5-point Likert scale, with 1 = heavily disagree and 5 = heavily agree. Data included in this study analyzes responses to 11 survey items across three topic sections:

(1) Trust in Police/Public: This section of the survey, comprising four prompts, aimed to assess the level of trust between the police and the public. An example of a prompt provided to participants is “Members of law enforcement follow the rules.”
(2) Interactions: Consisting of two prompts, this section assesses the public's opinion on interactions with officers. Prompts include “Members of the police treat the public with respect”.
(3) Cooperation: Lastly, participants were asked about cooperation with police, which heavily affects police effectiveness and efficiency (Bolger and Walters, 2019; Tyler, 2004). The prompts consist of four items, including “I would not hesitate in reporting potential crimes to the police.”

The final dataset included 640 females (61%) and 410 males (39%). The sample tended to be relatively young, with a mean age of 30.1 years (SD = 10.3), although males were slightly older on average (32.1 years) than females (28.8 years). The ages ranged from 18 to 75 years, encompassing multiple generations. Regarding marital status, the sample was nearly evenly split, with 52% of participants single and 48% married. Educational attainment was notably high, with 76% of participants having completed education beyond high school (associate's degree or college). Geographically, participants were distributed across six governorates within Kuwait, with the largest groups from Farwaniya (16%), Mubarek (15%), and Ahmadi (16%). Smaller groups came from Asma (5%), Hawalli (6%), and Jahra (9%). Complete descriptive statistics are presented in Table 1 below.

**Table 1:** Descriptive statistics for survey participants (by gender).

	**Female (*N =* 640)**	**Male (*N =* 410)**
Variable:	*n* (cumulative %)	
**Age:**		
Female mean (SD): 28.82 (9.85) Median (min, max): 26 (18, 75) Male mean (SD): 32.10 (10.71) Median (min, max): 30 (18, 65)		
**Social status:**		
Single	357 (56%)	191 (47%)
Married	283 (44%)	219 (53%)
**Job status:**		
Employed	280 (44%)	261 (64%)
Retired	28 (4%)	40 (10%)
Student	284 (44%)	93 (22%)
Unemployed	4 (8%)	16 (4%)
**Education:**		
> High school	536 (84%)	262 (65%)
≤ High school	104 (16%)	148 (35%)
**Province:**		
Ahmadi	148 (23%)	94 (23%)
Asma	55 (9%)	25 (6%)
Farwaniya	152 (24%)	90 (22%)
Hawalli	46 (7%)	53 (13%)
Mubarek	132 (21%)	101 (25%)
Jahra	107 (17%)	47 (12%)

Kuwait's National Police Force fully approved the use of data in this study. Data collection was conducted in Kuwait's native language, Arabic. The datasets used included no identifiable information related to participants.

### 2.2 Analytical strategy

After carefully assessing the datasets, it became clear that pre-diagnostics were necessary to address missing data in survey responses. Multivariate imputation by chained equations (MICE) was employed for this purpose. In both datasets, the percentage of missing data ranged from about 1% to 17%, with no variables exceeding a threshold that would threaten the validity of the imputation model. Because the overall missingness rate and pattern aligned with the missing at random (MAR) assumption, multiple imputation was deemed an appropriate and reliable method. A total of 20 imputations were performed, allowing for pooled estimates in the subsequent statistical analysis (see White et al., 2011).

Two primary data analysis methods were identified to align with the research objectives posed in this study. The first involves a preliminary pairwise analysis to identify significant differences in opinions between the two gender groups. Mann-Whitney tests were performed on the survey topics to establish significant differences.

Secondly, multivariate testing was used to identify factors related to sentiments among women, offering valuable insights into demographic elements that potentially correlate with their sentiments. These steps establish a strong and comprehensive analytical strategy that can effectively address the research questions posed in this study. Pre-diagnostics in the form of outlier removal (Cook's D) and variance inflation checks (VIF) were used. Due to the error terms not being normally distributed, generalized linear models (GLMs) were used, employing the Gaussian family and identity link.

However, before conducting any analysis, it was necessary to perform tests of internal validity and to establish constructs for analysis using appropriate statistical methods. Confirmatory factor analysis (CFA) was employed to create composite variables for each of the three topic area items, yielding overarching measures that represent overall sentiments across the survey items within each area. Survey items for each section were grouped and subject to thresholds for factor loading (set at >.40), with a Kaiser criterion of 1.0 per Hair et al. (2022). These composite variables effectively represented each topic area included in survey data, serving as the outcome variables in our models. Full details of the factors and individual item loadings are available in Table 2.

**Table 2:** Confirmatory factor analysis for constructs.

**Construct**	**Survey item:**	**Factor loading**	**Eigenvalue**
**Trust:**			2.645
	I usually believe in the words of the police	0.817
	I believe that the motives of members of the police are always pure	0.858
	Members of the police follow the rules	0.665
	Members of the police respect the law	0.662
**Interactions:**			1.690
	Members of the police treat the public with respect	0.8453
	Members of the police are polite	0.8453
**Cooperation:**			2.546
	I would help members of the police if needed	0.682
	I would not hesitate in reporting potential crimes	0.797
	I would be open to providing testimony to law enforcement were it requested of me	0.710
	I would not hesitate asking members of the police for help	0.683

Included in the table are constructs representing sentiments, survey items, factor loadings, and eigenvalues for constructs.

Lastly, it should be noted that factors not serving as outcome variables in their respective multivariate models will be used as controls, since the literature heavily indicates intersections between them. Using them as controls will enhance the models' explanatory power, providing a more robust analysis of our main predictors, as represented by the collected demographics.

## 3 Results

### 3.1 Pairwise testing

CFA confirmed three main sentiment constructs: Trust (eigenvalue = 2.645), Interactions (eigenvalue = 1.690), and Cooperation (eigenvalue = 2.546), indicating their suitability as factors for analysis (see Table 2). At the construct level, significant gender differences emerged for Interactions (*p* = 0.0314) and Cooperation (*p* = 0.0049), while Trust showed no significant difference (*p* = 0.0797).

This construct comprised four items with factor loadings ranging from 0.662 to 0.858, including beliefs about police words (λ = 0.817), pure motives (λ = 0.858), following rules (λ = 0.665), and respecting the law (λ = 0.662). For the Trust construct, males and females showed similar overall sentiment (males M = −0.075, females M = 0.048), indicating no significant difference in opinions between the two genders.

The interactions construct included two items with high factor loadings: police treating the public with respect (λ = 0.845) and police being polite (λ = 0.845). It revealed that females reported slightly more positive perceptions (M = 0.055) compared to males (M = −0.086), reaching statistical significance (*p* < 0.05).

**Figure 1:**
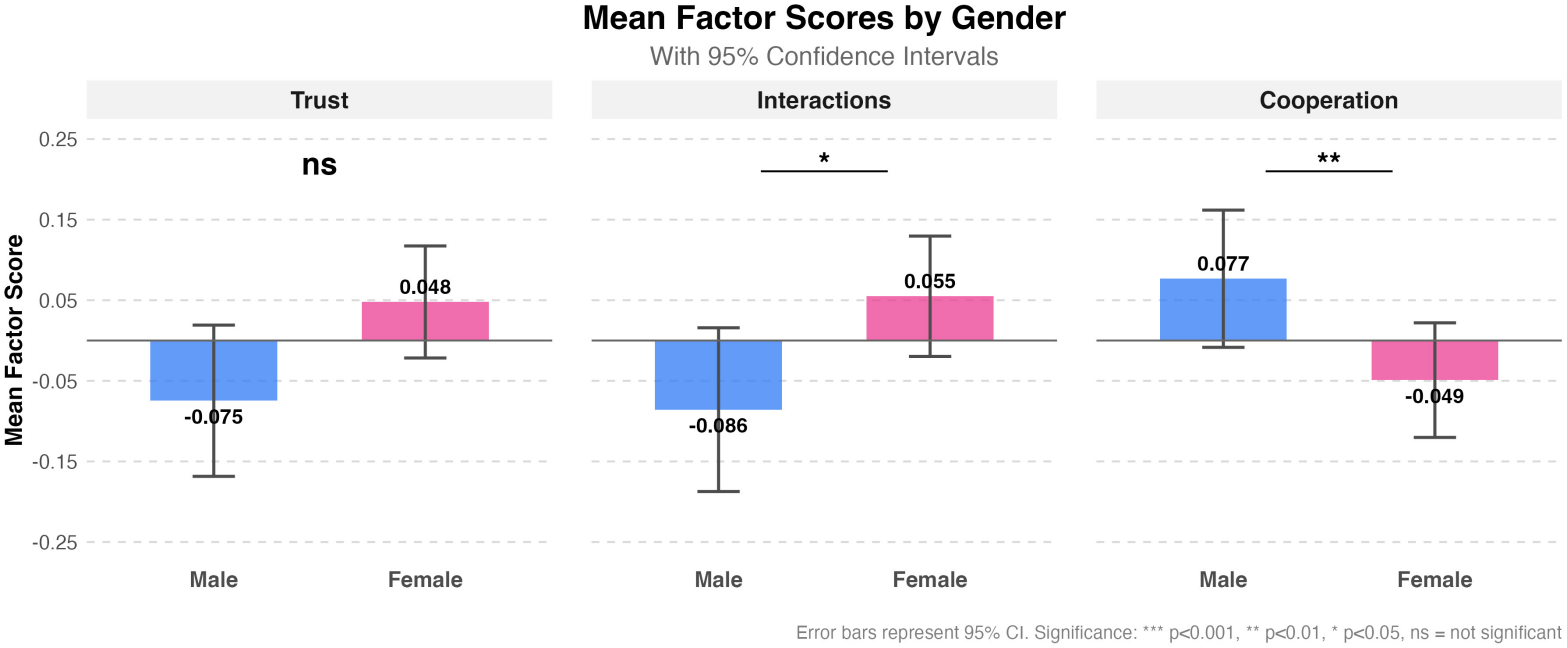
Gender-based differences. This visualization presents a comparative analysis of mean factor scores between males and females across three psychological constructs: Legitimacy, Citizenship, and Cooperation.

The cooperation construct comprised four items: helping police if needed (λ = 0.682), reporting potential crimes (λ = 0.797), providing testimony to law enforcement (λ = 0.710), and asking police for help (λ = 0.683). Males demonstrated a significantly higher willingness to cooperate (M = 0.077) than females (M = −0.049). Figure 1 visualizes all pairwise analyses.

### 3.2 Multivariate testing

For multivariate analysis, age (in years), marital status (1 = married), educational background (1 = associates/college), job status (reference: employed), and province of residency (Asma, the capital province, serving as reference). Detailed results are presented in Table 3 below.

**Table 3:** Multivariate models predicting females‘ sentiments on the police.

**Predictor**	**Trust**	**Interactions**	**Cooperation**
**Demographics**			
Age	0.001 (0.004)	−0.006 (0.003)	0.011 (0.004)^*^
Social (married)	0.097 (0.060)	0.016 (0.057)	−0.135 (0.068)^*^
**Job status:**			
Retired	−0.049 (0.136)	0.225 (0.128)	−0.211 (0.152)
Student	0.121 (0.064)	0.012 (0.061)	0.010 (0.074)
Unemployed	0.011 (0.105)	0.028 (0.100)	0.118 (0.122)
Education	0.004 (0.068)	0.159 (0.064)^*^	−0.129 (0.076)
**Province:**			
Ahmadi	−0.077 (0.092)	−0.090 (0.088)	−0.034 (0.107)
Farwaniya	−0.078 (0.066)	0.062 (0.063)	0.017 (0.076)
Hawally	−0.172 (0.100)	0.020 (0.096)	0.115 (0.114)
Jahra	−0.064 (0.068)	−0.061 (0.065)	0.125 (0.079)
Mubarek	−0.022 (0.073)	−0.048 (0.069)	−0.038 (0.084)
**Factor scores**			
Trust	-	0.649 (0.027)^***^	0.226 (0.041)^***^
Interactions	0.708 (0.028)^***^	-	0.231 (0.042)^***^
Cooperation	0.126 (0.028)^***^	0.140 (0.027)^***^	-
*n*	614	605	609
AIC	1,046.35	958.1	1,205.35
BIC	1,112.65	1,024.18	1,271.53
R2	0.623	0.616	0.265

The following table exhibits results from a multivariate analysis predicting Kuwaiti females' opinions of law enforcement in Kuwait. It utilizes demographic and sentiment-related predictors to achieve this. Goodness of fit measures are also reported (n, AIC/BIC, and R2).

Social status (1 = married); Job Status (ref = employed); Education (1 = Associates/College); Province (ref = Asma).

Standardized beta coefficients; *standard error* in parentheses.

^*^p < 0.05, ^**^p < 0.01, ^***^p < 0.001.

The model predicting women's Trust showed strong explanatory power (R^2^ = 0.623). None of the demographic or geographic predictors reached statistical significance for this outcome. However, the cross-relationships with other police perception variables were substantial: Interactions showed a strong positive relationship (β = 0.708, *p* < 0.001), and Cooperation also positively predicted Trust (β = 0.126, *p* < 0.001).

The interaction model demonstrated a good model fit (R^2^ = 0.616). Educational background (associates/college) positively predicted Interactions (β = 0.159, *p* < 0.05), indicating that females with an associates or college education held more positive views about police interactions compared to those with a high school education. Among the geographic variables, none showed a significant relationship. The model revealed strong positive cross-relationships with Trust (β = 0.649, *p* < 0.001) and Cooperation (β = 0.140, *p* < 0.001).

The cooperation model had the lowest explanatory power (R^2^ = 0.265) but revealed several significant predictors. Age positively predicted cooperation willingness (β = 0.011, *p* < 0.05), suggesting older women were more willing to cooperate with the police. Conversely, married women showed lower cooperation tendencies (β = −0.135, *p* < 0.05). The model showed positive cross-relationships with both Trust (β = 0.226, *p* < 0.001) and Interactions (β = 0.231, *p* < 0.001).

## 4 Discussion

### 4.1 Gender-based differences in opinions

The primary research question posed in this study centers on how culturally based gender norms and inequality shape opinions surrounding the police and women's acceptance toward contacting them. To do so, pairwise comparisons were conducted between men and women to identify significant differences in their opinions on various themes related to policing. These topics extended to issues like trust in the police, police behavior, and willingness to cooperate. Significant differences were found between our groups (Figure 1), indicating that men and women have fundamentally different and unequal experiences with law enforcement.

According to the results, women often had more favorable opinions of law enforcement in Kuwait, responding more favorably to items that painted police personnel in a favorable light (e.g., Members of the police treat the public with respect). However, the reverse was exhibited for items discussing their willingness to cooperate with police, with females being significantly less likely to cooperate. These findings show a nuanced perspective that reveals the complex and often contradictory ways gender ideology operates in Kuwait.

Potential explanations for these findings can be found in past literature. Scholarly work on the subject found women were more likely to have favorable views on law enforcement due to their being more likely to have fears regarding crime and victimization, something that reinforces views of law enforcement being protectors (Warr, 1984). Furthermore, studies have found that women supported law enforcement more (Hitchens et al., 2023) and community-oriented policing (Lee et al., 2019). It should be noted, however, that the literature on this varies. For instance, Wilson et al. (2021) found that women have more negative perceptions of police, while Brown and Reed Benedict's (2002) meta-analysis indicated contrasting literature on the relationship. Also relevant are law enforcement's opinions of women within Kuwaiti society, particularly their capacity to commit crimes. Women rarely commit crimes in Kuwait, an element that makes male officers less vigilant and suspicious around them when compared to their male (see Farrell, 2015).

According to research by Avdija (2010) and Novich et al. (2018), the lenient treatment that women often receive from police contributes to unequal outcomes and shapes gendered differences in opinion. A potential explanation as to why Kuwaiti men have starkly more negative opinions of officers' behavior when compared to females is that they are subject to different, and often more suspicious, forms of policing. These trends are often attributed to paternalistic views, which, while potentially leading to less hostile encounters for women, reinforce gender stereotypes rather than uphold the principle of equal treatment for all citizens (Carrillo, 2021).

Notably, significant differences were found in women's opinions on cooperating with police, highlighting females' lower tendency to engage with law enforcement, even in cases where crimes were committed. This critically important finding highlights how positive personal interactions do not necessarily translate into increased cooperation by women with police in the Middle Eastern context, underscoring the influence of cultural dynamics on this outcome. This strongly contrasts Western literature finding positive sentiments equating to increased cooperation (review Bolger and Walters, 2019).

Our findings suggest that Kuwaiti women's reluctance to engage with police is not an isolated phenomenon, but rather one facet of a broader regional pattern of gendered institutional avoidance. For instance, Salem and Yount (2019) exploration of desegregation in the Qatari workforce revealed complex schemas pertaining to gender dynamics in the workplace, one prioritizing the protection of females' reputation through minimizing interactions with men. Maintaining familial ties and marriageability play a catalytic role in subscribing to historic patriarchal structures.

Similarly, in the business world, research by Megheirkouni et al. (2020) provided insight into the lengths Arab women go to appease societal expectations tied to traditionalism. Their research argues that Arab women possess strong entrepreneurial intent; however, they are significantly inhibited by cultural constraints when networking or collaborating with men, adversely shaping women's professional interactions and prospects, something corroborated in Koburtay et al. (2020) work in Jordan. Just as women in Qatar and Jordan navigate professional barriers, women in Kuwait face cultural barriers to justice.

Kuwait's police force is predominantly made up of men, with female officers only being allowed to join the force in 2009. Even with their entry, they form an overwhelming minority within the police (potentially no more than 2%). This discrepancy reinforces the profession of law enforcement in Kuwait as a male-dominated profession. This could explain women's reluctance toward cooperating with the police, knowing that interactions with them will most likely include ones with men, creating a direct conflict with powerful social norms.

This reluctance is not merely a preference but a direct reflection of structural barriers. Even in Kuwait, a country considered one of the most liberal among the GCC, women are often heavily discouraged from visiting criminal justice institutions (e.g., police stations), and if they do, only in the attendance of a male family member or spouse. This finding has a significant implication: it provides empirical evidence that women often feel encouraged or compelled to delegate interactions with the state to male relatives. This effectively positions men as necessary components in achieving justice for women, filtering their access to protection and legal recourse. Such a dynamic, where a man may mediate a woman's access to the state, is a clear and tangible barrier to justice, representing a foundational aspect of gender inequality within Kuwaiti society.

### 4.2 Determinants of female opinions

Beyond the general differences between men and women, our findings reveal how specific characteristics influence women's perceptions and willingness to engage with the police. The multivariate analysis shows that education level predicts women's views on police behavior, while age and marital status are significant determinants of their willingness to cooperate. These factors highlight how the experience of gender inequality is not uniform, but is shaped by a woman's social position and life stage.

Our finding that higher education is associated with more positive views of police behavior highlights the mediating role of education in state-citizen interactions. Education may serve as a factor influencing perceptions of police because it equips women with the social and cultural capital to navigate interactions with male authority figures and institutional systems more confidently (Wentz and Schlimgen, 2012). Arguments can be made that educated women are often more likely to challenge traditional gender roles and less likely to find interactions with law enforcement discomforting (Auletto et al., 2017; Thomas and Kasselstrand, 2022). Lastly, educated women might better understand their rights when coming into contact with law enforcement, which in turn can reduce anxiety (see Dai et al., 2019). In this context, education serves as an empowerment tool, mitigating some of the social constraints imposed by patriarchal norms.

Perhaps even more revealing are the factors that predict women's willingness to cooperate. The finding that married women are less likely to cooperate with law enforcement provides strong empirical support for our hypothesis that males serve as selected representatives when dealing with the criminal justice system. In this traditional context, marriage may formalize the husband's role as the family's intermediary with public institutions, thereby increasing the social pressure on women to delegate such interactions. This result is highly indicative of a structural barrier where a woman's marital status corresponds with deference to spouses on issues related to the criminal justice system.

Conversely, the finding that older women are more willing to cooperate suggests that age may grant women a degree of social authority and autonomy that younger women lack. Older women, who may have raised families or been widowed, may be less constrained by the same reputational concerns and more confident in dealing directly with officers. Taken together, these demographic determinants paint a complex picture: factors like education and age can provide some women with the tools to navigate patriarchal structures, while marriage can reinforce the very barriers that produce gender inequality in access to the state.

### 4.3 Limitations

Like any study, this research faces several limitations, both specific to this study and common to survey-based research. A primary limitation of this study is its sampling strategy. Convenience and snowball sampling, initiated through university networks, introduces a clear bias toward a younger, more educated, and urban demographic. This methodological choice means our findings, while internally valid, cannot be generalized to the entire Kuwaiti population. The perspectives of older and less-educated women, who may face different or stronger cultural barriers, are likely underrepresented. Therefore, the results should be interpreted as reflecting the views of a specific, more socially connected segment of the population, rather than a national consensus. Future studies should use more robust sampling methods that guarantee representation across Kuwait's diverse regions and social groups, including those less likely to participate in academic networks.

Cultural factors unique to Kuwait and, more broadly, the Middle East also restricted the range of topics and themes the researchers could explore. Compared to Western societies, individuals in the Middle East may be less inclined to participate in surveys, especially when questions are perceived as too personal, sensitive, or potentially problematic. This hesitancy can extend to providing demographic data related to socioeconomic status, ethnic or tribal background, and political views—possibly contributing to the low effect sizes observed in the study's models.

Additionally, while this research addresses gaps in Middle Eastern literature on police-public relations, it is important to consider that Kuwait is only one country within the region, with distinct cultural, societal, and legal dynamics that may differ significantly from those of Arab neighbors, particularly regarding patriarchal systems. This uniqueness affects the study's generalizability, highlighting the need for comparative studies across multiple Middle Eastern contexts to strengthen insights into regional police-public dynamics.

## 5 Conclusion

This study represents a pioneering effort to explore gender and public perceptions of law enforcement in the GCC, providing valuable insights into the cultural and societal dynamics unique to the Middle East and Arab world. By examining gender dynamics, cultural norms, and public sentiment toward law enforcement, the research underscores the distinct influence of patriarchal traditions, concepts of honor, and societal values on interactions with the police. This article makes a significant contribution to understanding the factors shaping police-public relations in the region, addressing critical gaps in the literature on criminal justice in the Arab world. Moreover, the study emphasizes the importance of implementing targeted reforms and policies that take into account these cultural nuances to enhance trust and cooperation between the public and law enforcement in the Middle East.

To address the deeply ingrained norms identified in this study, Kuwait should pursue comprehensive, gender-responsive policing reforms. These reforms should include a strategic recruitment drive to significantly increase the number of female officers at all levels, lowering the social barrier for women to report crimes and cooperate with police. Critically, this must be paired with creating female-centric reporting mechanisms, such as dedicated hotlines or staffed women's desks in police stations. While such structural changes may face institutional inertia, they are essential for providing women with direct and autonomous access to justice.

Furthermore, these reforms would align Kuwait's policing practices with international standards. Specifically, they reflect the principles of UN Security Council Resolution 1325, which calls for the increased participation of women in all security efforts, and align with frameworks promoted by UN Women that emphasize creating safe reporting channels to ensure women's equal access to state protection. By implementing these measures, policymakers can take concrete steps to promote women's genuine empowerment and well-being.

## Data Availability

The data analyzed in this study is subject to the following licenses/restrictions: Data used for this study is available for sharing; however, access requires prior permission. Interested researchers can request access to the data by contacting the corresponding author. All requests will be reviewed and are subject to approval by Kuwait's National Police. Requests to access these datasets should be directed to nalsabah@jjay.cuny.edu.

## Footnotes

Edited by: Nayera Shousha, Doha Institute for Graduate Studies, Qatar

Reviewed by: Gokhan Savas, American University of Sharjah, United Arab Emirates Nesreen Hosny, Cairo University, Egypt
